# Use of Ultrasound to Verify the Impact of Telemedicine Myofunctional Therapy on Sleep Apnea Syndrome: Study Protocol Proposal

**DOI:** 10.3390/life14020197

**Published:** 2024-01-30

**Authors:** Cristina Rodriguez Alcala, Laura Rodriguez Alcala, Jose Maria Ignacio Garcia, Guillermo Plaza, Peter Baptista, Guillermina Lujan, Paula Mazzei, Juan Antonio Ibañez-Rodriguez, Carlos O’Connor-Reina

**Affiliations:** 1 Anesthesiology Department, Hospital Quironsalud Marbella, 29680 Marbella, Spain; cristina.rodriguez@quironsalud.es; 2 Otorhinolaryngology Department, Hospital Quironsalud Marbella, 29680 Marbella, Spain; laura.rodriguez@quironsalud.es (L.R.A.); mazzeipaulac@gmail.com (P.M.); juanantonio_ibanez_rodriguez@hotmail.com (J.A.I.-R.); 3 Otorhinolaryngology Department, Hospital Quironsalud Campo de Gibraltar, 11379 Palmones, Spain; 4 Neumology Department, Hospital Quironsalud Marbella, 29680 Marbella, Spain; 5 Otorhinolaryngology Department, Hospital Universitario de Fuenlabrada, Universidad Rey Juan Carlos, 28943 Madrid, Spain; guillermo.plaza@salud.madrid.org; 6 Otorhinolaryngology Department, Hospital Sanitas la Zarzuela, 28023 Madrid, Spain; 7 Otorhinolaryngology Department, Clínica Universitaria de Navarra, 31008 Pamplona, Spain

**Keywords:** ultrasound, myofunctional therapy, telemedicine, sleep apnea, sleep-disordered breathing, oropharyngeal exercises, drug-induced sleep endoscopy

## Abstract

The anatomy of the upper airways influences the risk of obstructive sleep apnea (OSA). The size of soft tissue structures, such as the tongue, soft palate, and lateral walls of the pharynx, contributes to the pathogenesis of OSA. New lines of treatment for sleep apnea, such as myofunctional therapy (MT), aim to strengthen the oropharyngeal musculature to improve the defining parameters of apnea. The present protocol uses ultrasound imaging to measure the size of the lingual musculature non-invasively and cost-effectively and evaluates the changes in its morphology. Eligible patients include those with OSA who have undergone submental cervical ultrasound and drug-induced sleep endoscopy before starting MT with the AirwayGym app. Follow-up evaluations are conducted at 3 months after beginning treatment. Patients diagnosed with OSA via questionnaires and polysomnography or respiratory polygraphy are evaluated anatomically and functionally using the Iowa Oral Performance Instrument, a tongue digital spoon, somnoscopy, and submental cervical ultrasound to assess their responses to the AirwayGym app. The lingual thickness (mm) and volume (cm^3^) and the distance between both lingual arteries (mm) are measured. The AirwayGym app helps users and therapists monitor the patient performance of MT. Incorporating submental ultrasound can be a useful non-invasive tool to evaluate OSA and MT.

## 1. Introduction

Obstructive sleep apnea (OSA) is one of the most predominant respiratory diseases affecting 6–9% of women, 6–20% of men in middle ages, and 1–3% of children. Higher body mass index (BMI), agedness, and male sex are the most significant risk factors [1,2,3].

The anatomy of the upper airways also influences the risk of OSA, and the size of soft tissue structures, such as the tongue, soft palate, and lateral walls of the pharynx, contributes to the pathogenesis of OSA [4,5].

Different etiologies suggested to explain OSA include a reduced muscular genioglossus tone, low hypoglossal nerve activity not allowing the pharyngeal muscle to keep an open airway, anatomical factors like tonsils and adenoid hypertrophy, cephalometric alterations such as mandibular and hyoid bone position and the length of the soft palate, obesity and neck fat, a low respiratory arousal threshold, and, finally, alterations in the ventilatory control drive “loop gain” [6]. Some authors have suggested that the main reason for this disease is the malfunctioning of the genioglossus muscle, which is the main upper airway’s dilating muscle [6,7].

Magnetic resonance imaging (MRI) can provide a detailed characterization of the tongue, including its fat content, which has been shown to correlate with the severity of OSA [8,9]. Reducing tongue fat has been demonstrated a key factor in improving the apnea–hypopnea index (AHI) in OSA patients [9]. New lines of treatment for this disease, such as myofunctional therapy (MT), aim to strengthen the oropharyngeal musculature to improve the defining parameters of apnea, such as snoring, daytime sleepiness, AHI, and values on the Iowa Oral Performance Instrument (IOPI) for sensorimotor issues [10,11,12].

Floppy structures involving the upper airways appear to result in a narrow and collapsible airway. However, the effects of parapharyngeal fat deposits at different levels on the severity of OSA are not clear. Drug-induced sleep endoscopy provides three-dimensional and dynamic evaluations of upper airway collapse under conditions that better approximate natural sleep than with an awake patient [13].

MRI is the most commonly used imaging method to measure volumes and obstruction in the upper airways. Recent studies have incorporated computed tomography with the patient under sedation [14] and ultrasound. Ultrasound is a non-invasive, low-risk, and cost-effective imaging modality that can also be used to assess the tongue. Submental ultrasound can be used to characterize the tongue in patients with OSA and has shown that an increased width and thickness of the tongue are associated with severe OSA [15,16]. Ultrasound can also be used to examine the dynamic movements that characterize changes in tongue morphology with activities such as the Müller maneuver [17,18]. Yu et al. [19] reported that the ultrasound echo intensity increases with increasing fat content in the tongue and strongly correlates with tongue fat measurements in MRI.

In the context of OSA, submental ultrasound can be used to determine whether the size of the tongue muscles is adequate to keep the upper airways open during sleep. Small or weak muscles may increase the risk of airway obstruction and sleep apnea [20]. MT can increase the size and strength of the genioglossus and geniohyoid muscles, which helps to improve upper airway function and reduce the severity of OSA.

Myofunctional therapy (MT) is based on oropharyngeal exercises that try to obtain a proper tongue positioning in the oral cavity. Guimaraes [21] in his randomized clinical trial (RCT) demonstrated that oropharyngeal exercises reduced the OSAS severity from an apnea–hypopnea index (AHI) of 22.4 +/− 4.8 to 3.7 +/− 8.5 events/hour. MT is composed of isotonic and isometric exercises that target oral structures (i.e., the lips and tongue) and oropharyngeal structures (i.e., the soft palate and lateral pharyngeal wall). There has been an increasing number of studies evaluating the effect of MT in the form of case studies, case series, and, most recently, two RCTs [12,22,23,24,25,26]. Because MT raises serious concerns relating to adherence, our sleep unit has designed the AirwayGym app to perform these exercises.

Using this protocol, we can examine the usefulness of ultrasound in predicting the treatment response to MT delivered by specialists using the AirwayGym application employed in our sleep unit.

The AirwayGym application was created in 2018 by the Sleep Units of the Quiron Salud Marbella and Campo de Gibraltar Hospital together with the computer engineering departments and developed by the apnea company Bye.

The novelty of this application is that the patient carries out his therapy from home with his smartphone, providing him with feedback on the effectiveness of the exercises that can also be supervised by his therapist. Complete information about the application is provided at https://airwaygym.App/ (accessed on 27 January 2024). Every therapist can sign up and track their patients [27].

The objective is to conduct a prospective, non-randomized pilot study in patients with moderate–severe obstructive sleep apnea–hypopnea syndrome (OSAS).

We measure with ultrasound the thickness of the lingual and pharyngeal walls, the distance between both lingual arteries, and the height and lingual volume in the patients included in this study.

The desired impact is to improve adherence to CPAP and myofunctional therapy, as well as perform simple anatomic evaluations such as ultrasound. If this improves treatment adherence and can diagnose and treat these patients in the future, there is ample scope for further clinical trials.

## 2. Experimental Design

This is a nonsponsored prospective, non-randomized pilot study coordinated by the Pulmonology, Anesthesiology, and Otolaryngology Departments of Quirónsalud Marbella Hospital and Campo de Gibraltar Hospital, Andalucia, Spain. This study has been registered (ISRCTN92645461) and approved by the local ethics committee (AWGAP20232).

### 2.1. Study Population

This study will include patients diagnosed with sleep apnea–hypopnea at the participating centers and who agree to participate in this study.

### 2.2. Study Groups

The control group will include patients with recent moderate–severe sleep apnea (1–3 months) undergoing treatment with continuous positive airway pressure (CPAP) therapy. An initial ultrasound measurement of the tongue genioglossus fat will be made and 3 months after inclusion in this study.

The MT group will include patients with moderate apnea. MT exercises alone, once a day for 3 months, will be performed exclusively via the AirwayGym app. An initial ultrasound measurement and drug-induced sleep endoscopy (DISE) will be performed with repeated control at 3 months.

**Figure 1:**
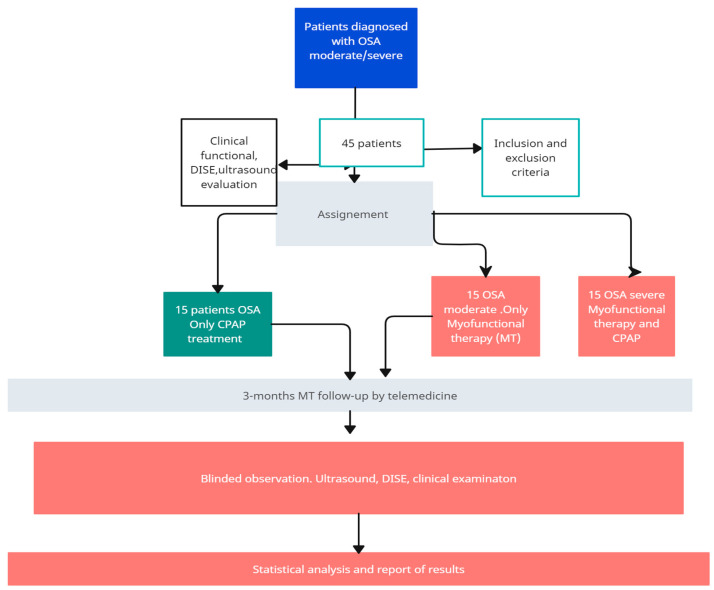
Study groups.

In the MT-CPAP severe apnea group, patients will use CPAP at night, measuring the number of hours they use it at night. A new sleep study will be performed at 3 months. An initial ultrasound measurement and DISE will be made with control at 3 months (Figure 1).

### 2.3. Objectives

- To evaluate the influence of MT on the reduction in cervical fat and the improvement of ultrasound parameters in sleep apnea.
- To measure different parameters related to the function and size of the genioglossus and geniohyoid muscles to evaluate the presence and severity of sleep apnea, as well as the effectiveness of myofunctional therapy in improving the function of these muscles.
- To determine whether performing a neck ultrasound is relevant for the indication of TM in sleep apnea.
- To evaluate whether performing ultrasound and DISE modify the indication of surgical techniques compared with if we only performed DISE.
- To evaluate the adherence to CPAP and MT.

The hypothesis of our work is based on determining whether knowledge of the fatty and muscular components can be decisive in the choice of pharyngoplasty techniques, in multilevel surgery, and as a possible predictor of response or failure to MT since, currently, the influence that TM can have on reducing cervical fat is unknown.

Given that the aforementioned tools only measure strength, we consider the previous study of cervical fat using ultrasound and its response to TM relevant in the study of collapse patterns during DISE.

## 3. Materials and Equipment

To develop the protocol, patients with sleep apnea were included who underwent submental cervical ultrasound and DISE before starting the AirwayGym App and after 3 months of treatment.

The measurements were made with the convex probe of the FUJFILM Sonosite LX ultrasound.

In submental ultrasound in the context of sleep apnea, different parameters can be measured to evaluate the size and function of the genioglossus and geniohyoid muscles, which are important for keeping the upper airway open during sleep. Some of the parameters that can be measured in submental ultrasound are:

1. Genioglossus and geniohyoid muscle thickness: The thickness of the genioglossus and geniohyoid muscles can be measured at different points on the lower part of the jaw. A smaller thickness may indicate weakness or atrophy of these muscles.
2. The cross-sectional area of the genioglossus and geniohyoid muscles: The cross-sectional area of the genioglossus and geniohyoid muscles can be measured at different levels of the lower jaw. A smaller cross-sectional area may indicate a decrease in the size of these muscles.
3. Tongue movement distance: The tongue movement distance forward from the rest position can be measured. A shorter movement distance may indicate weakness in the muscles that control the position of the tongue.

## 4. Detailed Procedure

Inclusion criteria (patients):

- Ages of 18–75 years;
- Diagnosis of moderate–severe OSA (AHI > 15);
- Not having used any previous treatment other than that in this study or lasting longer than 3 months in the case of the control group;
- Signature of informed consent (IC);
- Present good permeability and nasal function;
- Body mass index (BMI) of <30.

Exclusion criteria for both:

- Cognitive or neurological deficit;
- Inability to complete questionnaires;
- Severe alcoholism;
- Presence of craniofacial malformations;
- Active neoplastic disease;
- History of previous rehabilitation treatment of the orofacial musculature as well as any other treatment for previous apnea that could modify the results of this study (e.g., surgery or a mandibular advancement device (M));
- Temporomandibular joint dysfunction;
- Ankyloglossia of the tongue or inability to perform MT exercises;
- Not having a smartphone, or being unable to use it, or an Internet connection at home;
- BMI of >30;
- Weight changes with an increase of >5 kg during the 3 months of study.

### 4.1. Sample Size

Calculation of sample size

No studies have reported on the influence of MT on the reduction in cervical fat in patients with sleep apnea and evaluations of obstruction patterns in DISE before and after. Our study will initially be a pilot study, with 15 patients in the control group, 15 patients in the moderate MT apnea group, and 15 patients with MT-CPAP severe apnea.

### 4.2. Definition of Variables

The variables that we will measure in all patients and that are reflected in the data collection table using IBM SPSS Statistics version 28 with Windows software are as follows:

- Age;
- Sex;
- Weight;
- Height;
- BMI;
- Abdominal circumference (at the level of the navel);
- Neck circumference (with a flexible tape on the most prominent part; for this, it is necessary for the patient to be standing, with arms hanging at the sides, head erect, and gaze forward);
- Measurement with IOPI of tongue force and buccinator muscle;
- Measurement with digital spoon (DS);
- Initial AHI;
- Ranking of velum, oropharynx, tongue base, and epiglottis (VOTE) in DISE, before and after;
- Ultrasound measurements: echo intensity, tongue height (mm), and tongue width (mm).

To develop the protocol, patients with sleep apnea underwent submental cervical ultrasound and DISE before starting to use the AirwayGym app (www.airwaygym.app, accessed on 27 January 2024) and after 3 months of treatment.

**Figure 2:**
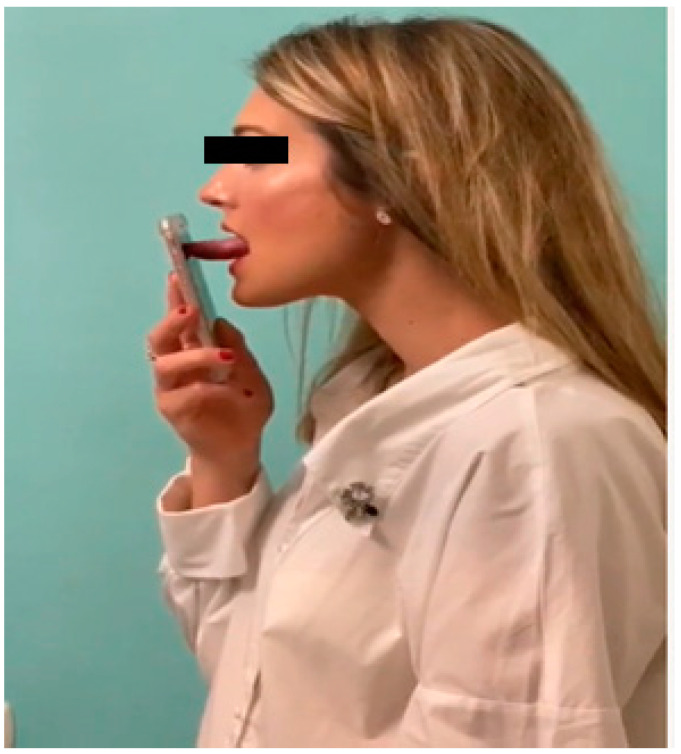
Exercise performed by a patient interacting with a smartphone screen via the AirwayGym app.

The AirwayGym app is a portable fitness app (Figure 2) intended for patients rather than athletes, and therapists rather than coaches provide the instructions. The novelty of this app is that it is the first healthcare app on the market that allows patients to directly interact with a smartphone without the need for any other device. The app focuses on improving proprioceptive deficits in patients with sleep apnea. When used with the app, the smartphone provides acoustic feedback on the effectiveness of the MT exercises performed [27].

The AirwayGym app comprises daily tension and concentration exercises (i.e., isometric and isotonic exercises, respectively) that can be performed anywhere. It offers the additional advantage of providing prompt feedback to allow patients to see their progress and resolve problems with the help of a therapist. Before each exercise, an animated demonstration and video provide instructions for performing the exercise. After each exercise, patients receive visual, acoustic, and tactile feedback as a score on the success of their performance. A chat function is available via which patients can contact the therapist directly. This app complies with regulations 2002/58/EC and (EU) 2016/679 regarding data protection. It is priced at EUR 8.99. Additional information can be found on the AirwayGym website.

The app presents a total of 9 myofunctional therapy exercises. Before each exercise, there is an explanatory video about them. The results of each of them are displayed with a percentage of success and are saved in an online network storage (in the cloud) and can be evaluated by a therapist. It also presents a virtual chat via which the user can communicate with a therapist.

The exercises are based on those described by Guimaraes in 2009 [21].

For hygienic reasons, we recommend covering the screen with a transparent film or cleaning the mobile screen with hydroalcoholic gel to perform the exercises [27,28].

Each of the exercises obtained from the AirwayGym website is explained below:

Exercise 1. Snake.

Stick your tongue out and press the screen with it for 5 s.

Exercise 2. Pressure with your chin.

Open your mouth and keep pushing the screen for 5 s.

While performing the exercise, say/a/.

Exercise 3. Chameleon up.

Stick out your tongue and press on the square at the bottom.

Exercise 4. Chameleon down.

Stick out your tongue and press on the square at the bottom.

Exercise 5. Tongue left cheek.

Press with the tip of your tongue the inside of the left cheek.

Contract the muscles of your cheek while pushing with the tongue toward the cheek.

Exercise 6. Tongue right cheek.

Press with the tip of your tongue the inside of the right cheek.

Contract the muscles of your cheek while pushing with the tongue towards the cheek.

Exercise 7. Pressure under chin.

Bend your head forward like you are going to drink from a cup and touch your chin on the phone.

Keep the contact and move your head from side to side for 10 s with your mouth closed.

Exercise 8. Left mandibular pressure.

Turn your jaw toward the phone and keep the pressure with your index finger on the screen for 5 s. Say/i/while performing it.

Exercise 9. Right mandibular pressure.

Turn your jaw toward the phone and keep the pressure with your index finger on the screen for 5 s. Say/i/while performing it.

**Figure 3:**
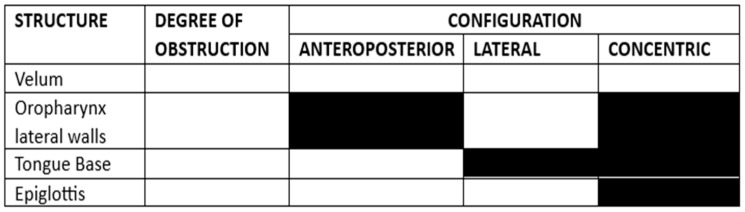
The surgical protocol used to code the DISE results according to the VOTE scale is described in reference [30].

DISE is performed in an operating room using a flexible endoscope and standard anesthetic monitoring of the oxygen saturation, electrocardiogram, and blood pressure. Although not mandatory, recording media (with or without audio) and playback equipment are recommended. DISE video sequences can be used for educational and research purposes or to inform patients about their test results. In our setting, an otolaryngologist will perform the endoscopic procedure, and an anesthesiologist will monitor the patients and observe their responses to medication and the procedure. Basic and advanced maneuvers (e.g., chin lift, jaw thrust, and head rotation) will be performed during DISE. The VOTE classification system is used to record the results [27,29,30,31] (Figure 3).

### 4.3. Assessments

Patients will be jointly evaluated by the pulmonology and otorhinolaryngology services of the hospital.

Evaluation by a pulmonology specialist: A detailed medical history is taken to record symptoms and risk factors associated with sleep apnea. BMI is calculated, and questionnaires that assess daytime sleepiness (the Epworth test; Appendix A) and sleep quality (the Pittsburgh questionnaire; Appendix B) are administered. Patients suspected of having sleep apnea undergo either polysomnography or diagnostic respiratory polygraphy based on their clinical symptoms. After this first assessment, the pulmonologist refers the patient to the otorhinolaryngology department to assess the anatomy and functionality of the upper airways. Patients with a BMI of >30 are transferred to the nutrition and dietetics unit. It is known that a reduction in BMI of 5–10% significantly reduces the AHI [32].

The sleep study is performed using standard polysomnography.

Standard nocturnal polysomnography involves (i) recordings of sleep-related electroencephalography (EEG), electromyography (EMG) of the chin and leg muscles, electrooculography (EOG), and electrocardiography (ECG); (ii) oxygen saturation; and (iii) measures of respiratory effort and airflow.

The tests are used to define PS with an AHI of <5 events/h of sleep, moderate OSAHS as an AHI of 15 to 29.9 events/h of sleep, and severe OSAHS as ≥30 events/h of sleep [eleven].

Laboratory-assisted polysomnography has been and continues to be a gold standard for the diagnosis of sleep-disordered breathing, although the usefulness of a single night-time recording for diagnosis can generate considerable variability in the AHI from night to night, especially when the AHI is low [11].

Therapy with the AirwayGym^®^ application will be initiated after inclusion in this study. This therapy will be applied to patients with an average duration of training of 5 days a week with 15–20 min/session. Three months after myofunctional therapy, an otorhinolaryngological evaluation, sleep questionnaires, and tongue strength measurements with the IOPI and ultrasound will be performed [11].

Evaluation by an otorhinolaryngology specialist: The otorhinolaryngology specialist is responsible for the anatomical and functional examination of the upper airways. During this consultation, the Friedman stage and Müller maneuver are assessed, and nasofibrolaryngoscopy is performed to determine the degree of retropalatal and/or retrolingual collapse. Subsequently, functional evaluation is performed using the IOPI Medical Device to assess tongue strength by measuring the maximum pressure and tongue fatigue by measuring resistance. Low resistance values indicate a high tendency to fatigue, in which case patients would benefit from treatment modulation with the AirwayGym app.

Another instrument for measuring tongue tone is the tongue digital spoon (TDS), which has recently been applied for the diagnosis and evaluation of patients with sleep apnea and lingual hypotonia [33,34]. In addition to telemedicine follow-up, patients will be provided with a sleep diary for symptom control (Appendix C to evaluate the response and adherence to therapy with the AirwayGym app. After this consultation, the patient will be offered DISE and cervical ultrasound to optimize the selection of the current treatments for sleep apnea [35].

**Figure 4:**
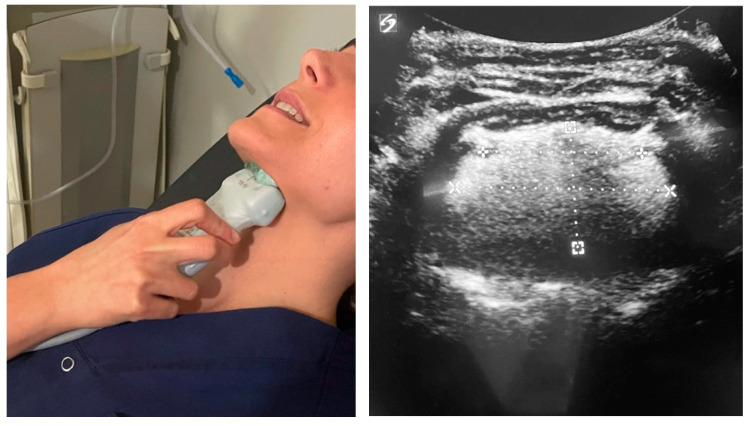
Performing a submental approach: coronal view.

**Figure 5:**
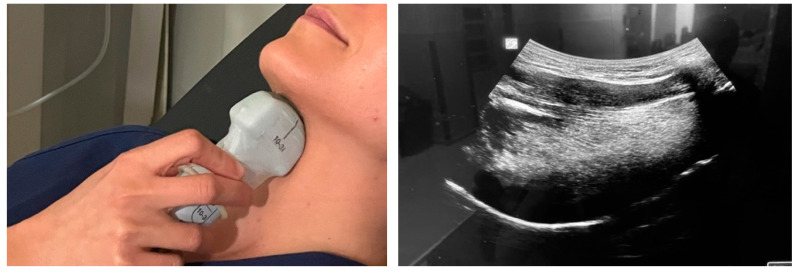
Performing a submental approach: sagittal view.

The distribution of visits to the center will be as follows:

- Pulmonology visit: Patients will be diagnosed with OSA in a pulmonology laboratory via an initial sleep study, which includes the measurement of baseline AHI, the nocturnal desaturation index, and the lowest oxygen saturation levels during the night.
- Otorhinolaryngology visit: Patients will be assessed by the otorhinolaryngologist, who will evaluate the anatomy and functionality of the upper airways using DISE and measure tongue strength with the IOPI and TDS. The AirwayGym app is recommended for patients with oropharyngeal hypotonia.
- Ultrasound visit: A neck ultrasound will be performed to measure tongue thickness (mm), height (mm), volume (cm^3^), and distance between the lingual arteries (mm) before DISE and at the start of treatment modulation with the app (Figure 4 and Figure 5).
- Monthly telemedicine follow-up: During the 3-month study period, all patients in the three groups will be monitored for adherence and incidents related to the prescribed treatment with the AirwayGym app.
- Three-month follow-up: All variables evaluated during the initial visit will be measured again after three months. The ultrasound visit is an evaluation added to this previously published protocol (Tongue +) [36]. A submental ultrasound will be performed in the context of sleep apnea.

The Kolmogorov–Smirnov test will be used for the distribution of quantitative variables. The chi-square test or Fisher test will be used as necessary for the study of categorical variables. Student’s *t*-test or ANOVA (for two or more samples) will be used to study the differences between quantitative variables.

Non-parametric tests (Mann–Whitney or Kruskal–Wallis) will be used if the variables to be analyzed do not follow a normal distribution. The level of statistical significance will be set at *p* < 0.

## 5. Expected Results

MT is a promising treatment for sleep apnea [37,38]. The AirwayGym app [39] is a mobile app for delivering MT using exercises aimed at strengthening the oropharyngeal muscles. Patients are instructed to perform the MT exercises for 20 min daily for at least 3 months. In most MT programs delivered without a smartphone, patients complete the exercises independently at home without feedback from the therapist. The main drawback of this type of MT is low patient adherence, which has been reported to be as low as 10% in some studies [40,41]. Baptista et al. [41] note that most existing mobile health apps for OSA have focused on the diagnosis of snoring or OSA. Smartphone technology can be valuable for treating people with OSA and for conducting MT.

When using this protocol, in addition to reinforcing the need for apps to promote adherence to MT in sleep apnea, we consider it necessary to clinically monitor the anatomical changes that occur during therapy because excess soft tissue surrounding the upper airways can contribute to a narrow and collapsible airway. However, the effects of parapharyngeal fat deposits at different levels on the severity of OSA remain unclear [20,21]. DISE provides accurate vision and evaluation of the site of the collapse of the upper airways under conditions that better approximate natural sleep than with the patient awake [18].

The objective of myofunctional therapy is to improve adherence to CPAP treatment since this monotherapy, despite its high effectiveness, has a low compliance rate.

Another objective set in this therapy is to carry out simple measurements for a better diagnosis and treatment [42].

Ultrasound is a non-invasive, low-risk, and cost-effective imaging modality that can also be used to assess the tongue. Submental ultrasound can be used to characterize the tongue in people with OSA and has shown that increased tongue width and thickness are associated with severe OSA. Ultrasound can also show dynamic movement that characterizes the changes in tongue morphology with activities such as the Müller maneuver [17,18,43]. Yu et al. [19] reported that ultrasound echo intensity increases with increased tongue fat content and strongly correlates with tongue fat measurements in MRI. Given that the IOPI and TDS measure only tongue strength, we consider it important to also measure cervical fat using ultrasound to clinically assess the responses to the AirwayGym app.

### 5.1. Preliminary Results

Additionally, submental ultrasound may be useful to evaluate the effectiveness of MT in sleep apnea. Studies have shown that myofunctional therapy can increase the size and strength of the genioglossus and geniohyoid muscles, which can improve upper airway function and reduce sleep apnea. Submental ultrasound can be used to measure these muscle changes and evaluate the effectiveness of therapy. With 10% of the patients included, there were significant differences (*p* < 0.05) in the initial volume (mm^3^) and lingual thickness (mm) between the control patients and the patients in the groups with moderate–severe sleep apnea. After 3 months of myofunctional therapy, we found a statistically significant improvement in the values of the functional examination with IOPI and the digital spoon and a reduction in lingual thickness (mm) of >15% in the patients studied.

### 5.2. Discussion

The purpose of this protocol is to establish a diagnosis and treatment model for patients with severe apnea. With new technologies, we can carry out personalized and targeted treatment using cost-effective and safe tools for our patients.

There are no publications on the influence of myofunctional therapy on the reduction in cervical fat in patients with sleep apnea and evaluations of obstruction patterns in DISE before and after. Our study will initially be a pilot, with 15 patients in the control group, 15 patients in the TM moderate apnea group, and 15 patients with TM-CPAP severe apnea. Adult obstructive sleep apnea syndrome (OSAS) that begins very early in life has been attributed to the abnormal development of anatomical structures that support the upper airway (UAS). This abnormal development may be related to genetic, epigenetic, environmental interactions, or all factors. There is a continuous interaction between normal orofacial development and orofacial motor functions, with the involvement of the sensorimotor innervation of the orofacial region, particularly the tongue and mouth. Myofunctional therapy is a theoretically effective treatment in patients with moderate OSAHS and an adjuvant treatment to continuous positive airway pressure therapy, but more scientific evidence is needed to establish its effectiveness. The hypothesis of our work is to study the sensorimotor function of the tongue (apraxia, strength, and stereognosis) before and after oropharyngeal exercises performed with an application based on proprioceptive rehabilitation called Airway Gym^®^ and to see if, in patients with sleep respiratory disorders that present abnormalities in the sensorimotor function of the UAS, there is a significant improvement after myofunctional therapy. Determining the knowledge of the fatty and muscular components can be decisive in the choice of pharyngoplasty techniques, in multilevel surgery, and as a possible predictor of response or failure to MT since, currently, the influence that MT can have on reducing cervical fat is unknown.

Given that the aforementioned tools only measure strength, we consider the previous study of cervical fat using ultrasound and its response to TM relevant in the study of collapse patterns during DISE.

Submental ultrasound is a non-invasive technique used to evaluate the size of the genioglossus and geniohyoid muscles, which are important in keeping the upper airway open during sleep. These muscles are located at the bottom of the jaw and are inserted into the tongue and larynx.

In the setting of OSA, submental ultrasound can be used to determine whether the size of these muscles is adequate to keep the upper airway open during sleep. If these muscles are small or weakened, there may be an increased risk of airway obstruction and sleep apnea [44]. Due to MT ameliorating OSA and sensorimotor deficit in selected patients, all clinical information obtained from the muscle pattern can be a useful tool for more accurate future therapeutic decisions [11,45].

We are interested in comparing the results obtained from the anthropometric characteristics of our patients with other populations to verify if there are significant differences between them [46,47].

Despite the strengths of this pilot study mentioned above, we note several limitations. First, the number of participants is small. The number of patients in the control group is low because we found significant differences in the therapy group early during this study. We therefore decided not to enroll more patients in the control group because of the difficulties experienced by patients with severe OSAHS not given appropriate therapy.

There is no blinding since it is a diagnostic protocol. Mood disorders are not analyzed since the exclusion criteria include patients with serious illnesses or those taking treatments that affect apnea.

## 6. Conclusions

The incorporation of digital apps to improve adherence to therapy is increasing. Our work focuses on developing a diagnostic and therapeutic protocol for treating sleep apnea using MT. The AirwayGym app helps users and therapists evaluate and follow up on the use and effectiveness of MT. In addition to DISE and instruments for measuring tongue strength (IOPI and TDS), the inclusion of submental ultrasound can provide a useful and non-invasive tool for evaluating sleep apnea and the responses to MT. We note that the submental ultrasound results should be interpreted by a trained and experienced professional when used in the context of sleep apnea.

## Data Availability

The data that support the findings of this study are available from the corresponding author (C.O.R.) upon reasonable request.

## Abbreviations

AHI	Apnea–hypoxia index
CPAP	Continuous positive airway pressure
IOPI	Iowa Oral Performance Instrument
mHealth	Mobile health
ODI	Oxygen desaturation index
OSAHS	Obstructive sleep apnea–hypopnea syndrome
BMI	Body mass index
VOTE	Ranking velum, oropharynx, tongue base ,and epiglottis
DISE	Drug-induced sleep endoscopy

## Appendix A

**Epworth Sleepiness Scale**

Name: ______________________________________________ Today’s date: _________________ Your age (Yrs): _______________ Your Sex (Male = M, Female = F): ________

How likely are you to doze off or fall asleep in the following situations, in contrast to feeling just tired?

This refers to your usual way of life in recent times.

Even if you haven’t done some of these things recently try to work out how they would have affected you.

Use the following scale to choose the most appropriate number for each situation:

0 = would **never** doze

1 = **slight chance** of dozing

2 = **moderate chance** of dozing

3 = **high chance** of dozing

***It is important that you answer each question as best you can***.

**Chance of Dozing (0–3)**

Sitting and reading

Watching TV

Sitting, inactive in a public place (e.g., a theatre or a meeting) _________

As a passenger in a car for an hour without a break _________________

Lying down to rest in the afternoon when circumstances permit ________

Sitting and talking to someone __________________________________

Sitting quietly after a lunch without alcohol ________________________

In a car, while stopped for a few minutes in the traffic ________________

## Appendix B

Pittsburgh Sleep Quality Index (PSQI)

Instructions: The following questions relate to your usual sleep habits during the past month only. Your answers should indicate the most accurate reply for the majority of days and nights in the past month. **Please answer all questions.**

1. During the past month, what time have you usually gone to bed at night? ___________________
2. During the past month, how long (in minutes) has it usually taken you to fall asleep each night? __________
3. During the past month, what time have you usually gotten up in the morning? ___________________
4. During the past month, how many hours of actual sleep did you get at night? (This may be different than the number of hours you spent in bed.) ___________________

## Appendix C

What time did you go to bed last night?
How long did it take to fall asleep?
What time did you wake up?
Did you wake up during the night? How often? For how long? Did you get up from bed?
How many hours did you sleep?
Yesterday, did you take any medications, including over-the-counter or herbal medications? What? When?
On a scale of 1 to 5, how tired are you? (very tired = 5)
In general, on a scale of 1 to 5, how tired were you yesterday? (very tired = 5)
On a scale of 1 to 5, did you have an unusual or stressful day yesterday? (very unusual or stressful = 5).
What did you do during the 30 min before going to bed?
How many hours do you use CPAP?
Did you do all the exercises?
Did you take a nap yesterday? How long? When?
Did you drink alcohol yesterday? How much?
Yesterday, did you have caffeine? How much? When?
Yesterday, did you do any physical activity? What? When?
Yesterday, did you eat a large meal or spicy food? What? When?
